# An Electrospinography Database of Gait-Related Tasks and Motor Imagery Exercises

**DOI:** 10.1038/s41597-026-07592-7

**Published:** 2026-06-09

**Authors:** Desirée I. Gracia, Eduardo Iáñez, Mario Ortiz, José M. Azorín

**Affiliations:** 1 Brain-Machine Interface Systems Lab, Miguel Hernandez University of Elche, Elche, 03202 Spain; 2 Engineering Research Institute of Elche - I3E, Miguel Hernandez University of Elche, Elche, 03202 Spain; 3 Valencian Graduated School and Research Network of Artificial Intelligence - ValGRAI, Valencia, 46022 Spain

**Keywords:** Spinal cord, Biomedical engineering

## Abstract

This study presents a dataset of electrospinography (ESG) signals recorded from the human spinal cord during gait-related activities and motor imagery tasks. The dataset was acquired as part of a broader initiative to develop a spinal-machine interface (SMI) for closed-loop control of lower limb exoskeletons. ESG signals were collected using high-density surface electromyography (HD-sEMG) electrodes from fourteen able-bodied participants performing baseline trials (2), movement execution tasks (12) and motor imagery tasks conducted both in static conditions (5) and during movement (5). The dataset encompasses multiple electrode configurations targeting the brachial and lumbar plexuses, as well as surrounding musculature, across three experimental protocols. A total of 10 sessions were recorded for Experiment 1 (one 64-electrode matrix), 10 sessions for Experiment 2 (two 32-electrode matrices) and 5 sessions for Experiment 3 (two-32 electrode matrices). Preprocessing techniques were applied to mitigate cardiac and motion artifacts. The data provides a valuable and pionneering resource for advancing neurorehabilitation research, allowing the refining of exoskeleton control strategies and improving artifact removal methods.

## Background & Summary

The project “ReGAIT - Development of a Self-Calibrated Neural-Machine Interface for Closed-Loop Control of Lower Limb Exoskeletons” focuses on the development of different neurorehabilitation techniques with the ultimate goal of integrating them into a novel motor rehabilitation system. This system employs both top-down and bottom-up approaches to optimize effectiveness, presenting a comprehensive solution for improving motor function in individuals with gait impairments.

This study focuses on the development of a spinal-machine interface (SMI) based on electrospinography (ESG) signals for closed-loop control of lower limb exoskeletons. The proposed interface holds significant promise for gait rehabilitation in patients with motor disabilities. To this end, the experimental protocol involved participants engaging in both gait-related tasks and motor imagery exercises. The insights derived from these recordings are expected to enhance the functionality of neurorehabilitation systems.

Spinal cord injury (SCI) is a debilitating condition that compromises mobility and independence due to damage to the nervous system. Traditional rehabilitation approaches often require intensive therapist involvement and yield inconsistent outcomes. The emergence of robotic exoskeletons offers a promising alternative to motor rehabilitation, as these devices can assist patients in regaining functional gait patterns^1^. The integration of brain-machine interfaces (BMIs) has further enhanced the potential of robotic systems by enabling direct patient control. Although brain signals recorded through noninvasive techniques such as electroencephalogram (EEG) have been extensively explored, researchers have sought to improve interface performance by combining EEG with other biosignals, as electromyographic (EMG) signals^2^. Expanding on these developments, this study explores the untapped potential of spinal cord signals. Beyond serving as a conduit for brain signals, the spinal cord independently processes information, offering a rich source of efferent signals that could revolutionize neural-machine interfaces (NMIs)^3^. By integrating spinal cord data with EEG signals, a more refined interpretation of user intent could be achieved, thereby enhancing the accuracy of exoskeleton control.

Spinal cord activity can be studied through direct neural recordings or indirect methods such as vascular flow analysis. Traditional recording techniques, though effective, are invasive and expensive. Fortunately, non-invasive and cost-effective alternatives are now within reach, including the use of high-density surface electromyography (HD-sEMG)^4^ and functional near-infrared spectroscopy (fNIRS)^5^. ESG derived from HD-sEMG, resembles traditional EMG recordings from the trunk as is susceptible to artifacts caused by electrocardiographic (ECG) activity. Although the precise frequency range of the ESG remains undefined, previous studies suggest that it spans from 0.5 Hz to 30 kHz^6,7^. However, this range overlaps with ECG components, which can extend up to 100 Hz and exhibit higher amplitudes^8^. Effective preprocessing is therefore essential to isolate spinal cord activity and maintain the fidelity of extracted signals.

The ESG dataset associated with this work represents a valuable resource for the research community. Despite decades of spinal cord studies, much of the focus has been on evoked potentials^9–11^, with comparatively limited attention devoted to movement-related activity and motor tasks, particularly those involving gait^10,12^. Furthermore, open ESG datasets remain scarce, and to our knowledge, no publicly available dataset includes ESG recordings acquired during both gait and motor imagery tasks. Existing resources also lack multi-configuration electrode recordings, limiting opportunities for comparative analyses of spatial resolution and electrode placement.

The dataset presented here addresses these gaps by providing ESG recordings from healthy participants across multiple experimental conditions, including baseline, gait execution and motor imagery. Importantly, it incorporates three electrode configurations: Experiment 1 employed a single 64-electrode matrix, while Experiments 2 and 3 used two 32-electrode matrices, targeting the brachial and lumbar plexuses as well as surrounding musculature. This multi-configuration design enables systematic evaluation of electrode layouts, signal quality and spatial coverage.

This work contributes to the growing body of literature by exploring ESG signals as a novel input for NMIs. The study establishes a foundation for refining robotic exoskeleton control and advancing gait rehabilitation through the investigation of both motor execution and motor imagery. As an initial step, ESG signals were recorded in healthy participants to characterize their features under controlled conditions. In addition, the dataset may facilitate the development of artifact mitigation techniques, particularly those targeting cardiac-induced noise. Overall, this dataset constitutes a pioneering resource for advancing spinal-based NMIs, improving exoskeleton control strategies and enhancing artifact-removal methods in neurorehabilitation research.

## Methods

This section describes the equipment used for data acquisition, the experimental protocols, participant details and data preprocessing techniques.

### Equipment

The ESG signals were acquired using HD-sEMG equipment operating at a sampling frequency of 2 kHz. Signal amplification was performed using the Sessantaquattro+ system (OT Bioelettronica, Italy), with data acquisition managed through OTBioLab+ software (OT Bioelettronica, Italy). The device was positioned on the participant’s back and secured with an adjustable strap, while a reference electrode was placed on the right scapula. To ensure optimal signal conductivity, the electrode application area was cleaned using a dermabrasive gel followed by ethyl alcohol.

**Fig. 1:**
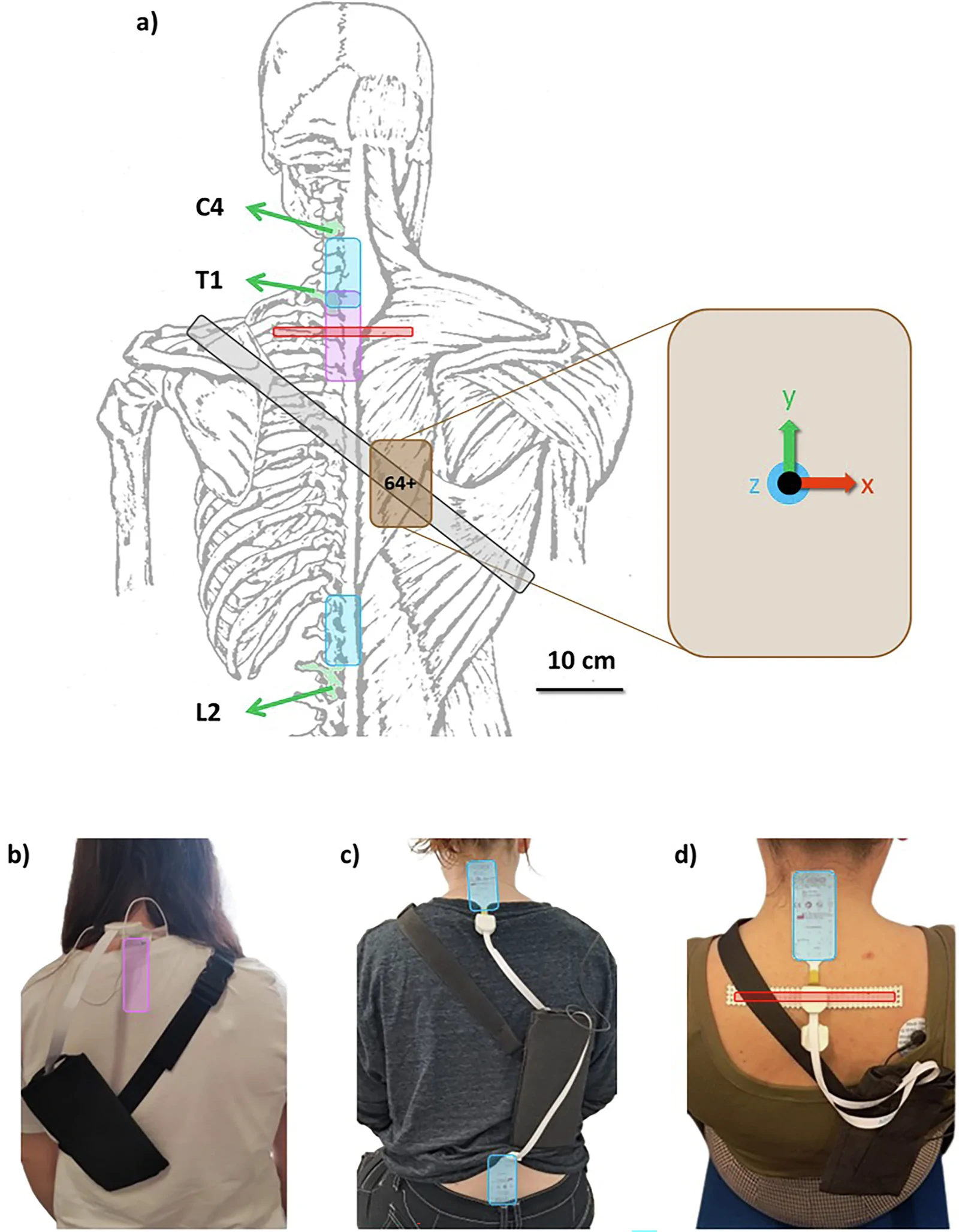
Subfigure **(a)** Placement of the various HD-sEMG matrix electrodes used in the three experiments, distinguished by colours corresponding to the three matrix configurations used. The vertebrae C4, T1 and L2, which served as anatomical fiducial points for electrode positioning, are shown in green. The figure also illustrates the placement of the Sessantaquatro+ device, secured with a band around the participant’s back, as well as the orientation of the axis used for the inertial measurements. Subfigures **(b),** **(c)** and **(d)** Photographic documentation of the matrix placement during the first, second and third experiments, respectively, employing the same color scheme to represent the different matrix configurations.

The dataset was compiled from three distinct experiments, each following a standardized protocol but employing different electrode configurations tailored to the specific research objectives. Fiducial points on the vertebrae (C4, T1 and L2) were identified through palpation of the spinous process to guide electrode placement along the midline of the spinal column, as illustrated in Fig. 1. The identification procedure followed established clinical guidelines, in which anatomically prominent and easily recognizable landmarks are first located and subsequently traced along the spinal column to reach the target vertebral levels^13^. In the case of the brachial plexus, the C7 vertebra was used as a primary reference, as it is typically the most prominent vertebra in the cervical region. When the participant is in a standing or seated position, C7 can be readily identified by flexion of the head and neck, which accentuates the most prominent protrusion at the posterior aspect of the neck corresponding to its spinous process. For the lumbar plexus, the procedure involved identifying the twelfth ribs and their attachment at the T12 vertebra, thereby establishing the position of its spinous process. A certain degree of human error in electrode placement is expected and accepted, as precise quantification of placement accuracy is not feasible without medical imaging. Furthermore, due to the fixed geometry of the electrode matrices and the natural anatomical variability across participants, electrodes positioned opposite the reference-aligned side of the array may rest at slightly different anatomical levels from one participant to another.

The experimental configurations were as follows:

- First Experiment: A matrix of 64 electrodes (1 mm diameter) arranged in a 13 × 5 configuration with an inter-electrode distance (IED) of 0.8 cm was employed to record activity from the brachial plexus. The matrix was positioned using the T1 vertebra as the anatomical landmark, with the first electrode aligned over the intervertebral disc located directly beneath the T1 vertebra.
- Second Experiment: Two electrode matrices, each with 32 electrodes (1 mm diameter) arranged in an 8 × 4 configuration with a 1 cm IED, were used. The first matrix was placed over the brachial plexus, using the C4 vertebra as a reference point, with its second electrode positioned over the intervertebral disc immediately below the C4 vertebra. The second matrix was placed over the lumbar plexus, using the L2 vertebra as the reference point, with its second electrode aligned over the intervertebral disc situated above the L2 vertebra.
- Third Experiment: Two electrode arrays were employed.

– An 8 × 4 matrix with 32 electrodes (1 mm diameter, 1 cm IED) was positioned using the C4 vertebra as the anatomical reference.

– A linear array consisting of 32 electrodes arranged in a 32 × 1 configuration (1 mm diameter, 0.5 cm IED) was placed a few centimeters below the lower edge of the first matrix, centered around the spine to capture spinal cord activity and the surrounding musculature.

Additionally, the Sessantaquattro+ system, secured with a band around the participant’s back, as represented in Fig. 1, simultaneously recorded quaternion data through the OTBioLab+ software at the same sampling frequency of 2 kHz. These measurements were acquired concurrently with the ESG signals to provide spatial orientation information.

### Protocol

**Fig. 2:**
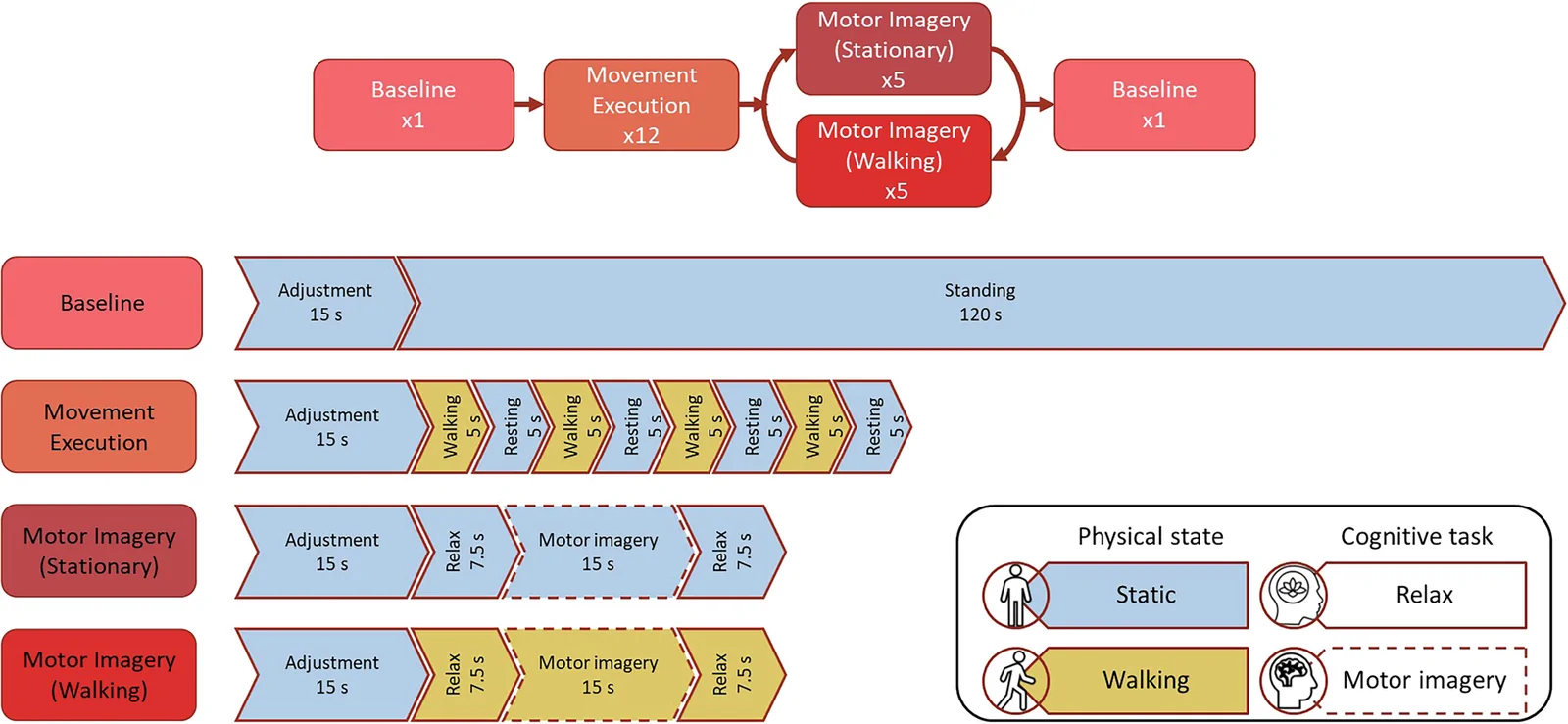
Representations of the four stages of the experimental procedure with their respective subdivision with tasks and times.

The experimental protocol was designed to capture ESG signals under three conditions: baseline (resting), active movement execution and motor imagery. The protocol consisted of four stages, as illustrated in Fig. 2.

- Baseline Recording (Stages 1 and 4, trials 1 and 24): At the beginning and end of each protocol, participants stood with their eyes open for two minutes to record resting-state activity.
- Active Movement Execution (Stage 2, trials 2–13): Participants alternated between walking and standing still, each task lasting five seconds. Task transitions were indicated by a one-second auditory cue and each cycle (walking and standing) was repeated four times. A total of 12 recordings were obtained during this stage.
- Motor Imagery (Stage 3, trials 14–23, with static and motion repetitions corresponding to even-numbered and odd-numbered trials, respectively): Participants alternated between periods of mental relaxation and kinesthetic motor imagery, consisting of 7.5 seconds of relaxation, followed by 15 seconds of motor imagery and concluding with another 7.5 seconds of relaxation. During the motor imagery task, participants imagined the sensations experienced in their lower limbs during walking. The tasks were cued by auditory signals and performed under both stationary and walking conditions, with each condition repeated five times, resulting in a total of 10 trials.

All experiments were conducted overground at a self-selected walking pace, without equipment assistance and participants wore comfortable footwear. Stage 2 primarily focused on transitions between motor states, particularly the initiation and cessation of gait. In contrast, Stage 3 emphasized motor imagery and enabled comparisons between continuous static and movement-related conditions.

To ensure that all participants engaged specifically in kinesthetic motor imagery, they were provided at the beginning of the experiment with detailed instructions outlining the different forms of motor imagery and emphasizing the characteristics of the kinesthetic modality. After receiving these instructions, participants were given time to practice the imagery task. They were then asked to verbally describe how they executed the task, allowing the experimenters to verify their understanding and to confirm that they were applying the kinesthetic strategy correctly.

To account for preprocessing and filter stabilization during data analysis, each recording session began with a 15-second relaxation period, during which participants remained still in a manner consistent with baseline recordings.

### Subjects

Fourten able-bodied subjects participated in the study. A total 10 sessions were recorded for experiment 1 (age: 30.30  ± 8.60, 40% female participants), 10 sessions for experiment 2 (age: 30.00  ± 8.99, 40% female participants) and 5 sessions for experiment 3 (age: 26.00  ± 4.24, 40% female participants). None of the participants reported any known diseases or movement impairments and all were right-footed. The participants were provided with information about the experimental procedure and signed a written informed consent form, as well as consent for video and image recording. The study was carried out in accordance with the Declaration of Helsinki of the World Medical Association and was approved by the Office of Responsible Research at the Miguel Hernández University of Elche (DIS.JAP.09.21).

The experiments were conducted over multiple days and not all participants engaged in every scenario due to scheduling constraints. Although this limits direct within-subject comparisons across the three experimental setups, the standardized protocol and consistent recording procedures allow for meaningful comparisons of the general trends associated with each electrode-matrix configuration. Table 1 presents an overview of participant involvement across sessions and provides an overview of their demographic characteristics.

**Table 1:** Sessions completed by each participant across the three experimental configurations, along with demographic information.

Subject	Experiment 1	Experiment 2	Experiment 3	Birth year	Gender	Height (cm)
S01	14/11/2023	23/05/2024		1997	Female	167
S02	15/11/2023	11/06/2024		1996	Female	160
S03	04/12/2023	07/05/2024		1998	Female	160
S04	05/12/2023			1996	Male	165
S05	18/12/2023	07/05/2024		1970	Male	170
S06	19/12/2023		04/10/2024	1991	Male	178
S07		27/06/2024 15/10/2024		1994	Male	167
S08			10/02/2025	2001	Male	175
S09			10/02/2025	2002	Female	160
S10	12/03/2026	11/02/2025	17/03/2026	1999	Male	178
S11	20/02/2025			1996	Female	160
S12	05/03/2026	09/03/2026		1992	Male	188
S13	05/03/2026	09/03/2026		2002	Male	172
S14		12/03/2026	13/03/2026	2002	Female	153

### Data preprocessing

The preprocessing pipeline was designed to enhance signal quality while preserving its integrity. Initially, a second-order Butterworth band-pass filter (10 Hz to 500 Hz) was applied to eliminate DC offsets and high-frequency noise. Power line interference at 50 Hz and its harmonics (up to 500 Hz) was mitigated using third-order Butterworth Notch filters. These filters were implemented as causal infinite impulse response (IIR) filters in state-space form and applied in a forward-only manner. Filtering was performed sample-by-sample with state propagation across overlapping 0.5 s segments to ensure continuity between segments. Consequently, this stage introduces the frequency-dependent phase shifts inherent to causal Butterworth filters.

Cardiac artifacts were addressed using the Adaptive Template Subtraction (ATS) algorithm, together with the preprocessing strategy proposed in^14^. This approach was selected for its denoising performance and low cutoff frequency, thereby minimally altering the properties of the original signal^15^.

The denoising sequence began with an R-peak detection algorithm to identify ECG peaks^16^. To further refine the signal, a sixth-order high-pass Butterworth filter with a cutoff frequency of 20 Hz was applied to remove motion artifacts and attenuate ECG contributions. For numerical stability, the filter was implemented in second-order-section form and applied using forward-backward zero-phase filtering, yielding zero phase distortion and an effective twelfth-order magnitude response. To reduce edge artifacts associated with IIR filtering, the signal was tapered at both ends using a Hann window prior to filtering.

The ATS algorithm was then applied independently to each channel to address residual cardiac artifacts. This method involved constructing an ECG subtraction template by averaging 40 ECG beats centered on the current beat. To ensure accurate alignment, the template was matched to the current beat using cross-correlation. The adjustment process was carried out separately for three distinct segments of the ECG waveform: the P wave, QRS complex and T wave. For each segment, linear regression was utilized to fine-tune the amplitude, offset and time scale. This iterative process was systematically repeated for all detected ECG beats, resulting in a cleaned signal with significantly reduced cardiac interference.

To accommodate diverse research requirements, the raw data were preserved, allowing database users to implement their own artifact removal methodologies if desired.

## Data Records

The data corresponding to the different electrode configurations is publicly available on the Zenodo platform^17^. Due to connection issues encountered during the file-saving process, four trials were saved incompletely. In the first experiment, trial 11 of subject S01 and trial 4 of subject S05, as well as trial 6 of subject S12 in the second experiment, contain approximately half a second less data than expected. Additionally, in the second experiment, trial 7 of subject S02 is truncated by four and a half seconds. Despite these incomplete recordings, the overall impact on the dataset is minimal, affecting at most a single repetition out of 48 for the same task. In addition to these partial losses, the same technical issue resulted in the complete loss of another three trials, accounting for less than 1% of the total number of trials. Specifically, trial 7 of subject S01 and trial 2 of subject S05 in the first experiment, as well as trial 9 of subject S07 in the second experiment are missing.

To facilitate transparent data usage, a machine-readable manifest file (*trial_integrity_manifest.csv*) is provided in the repository. This file lists all missing or truncated trials, including subject identifier, experiment number, date of acquisition, trial index, expected duration, actual duration and duration discrepancy.

### Repository

The datasets are publicly available on Zenodo^17^.

### Data Format

The data files are grouped into zip archives, with each archive corresponding to a specific experiment and subject. The naming convention for the zip files follows the format: “ExperimentX_SXX_YYYYMMDD”, where:

- “ExperimentX” denotes the experiment number (1, 2 or 3).
- “SXX” represents the participant code.
- “YYYYMMDD” specifies the experiment date.

Each zip file contains 24 MATLAB files, one for each trial. The individual “.mat” files follow the naming convention: “SXX_YYYYMMDD_Trial_XX”, where “Trial_XX” ranges from 1 to 24.

**Fig. 3:**
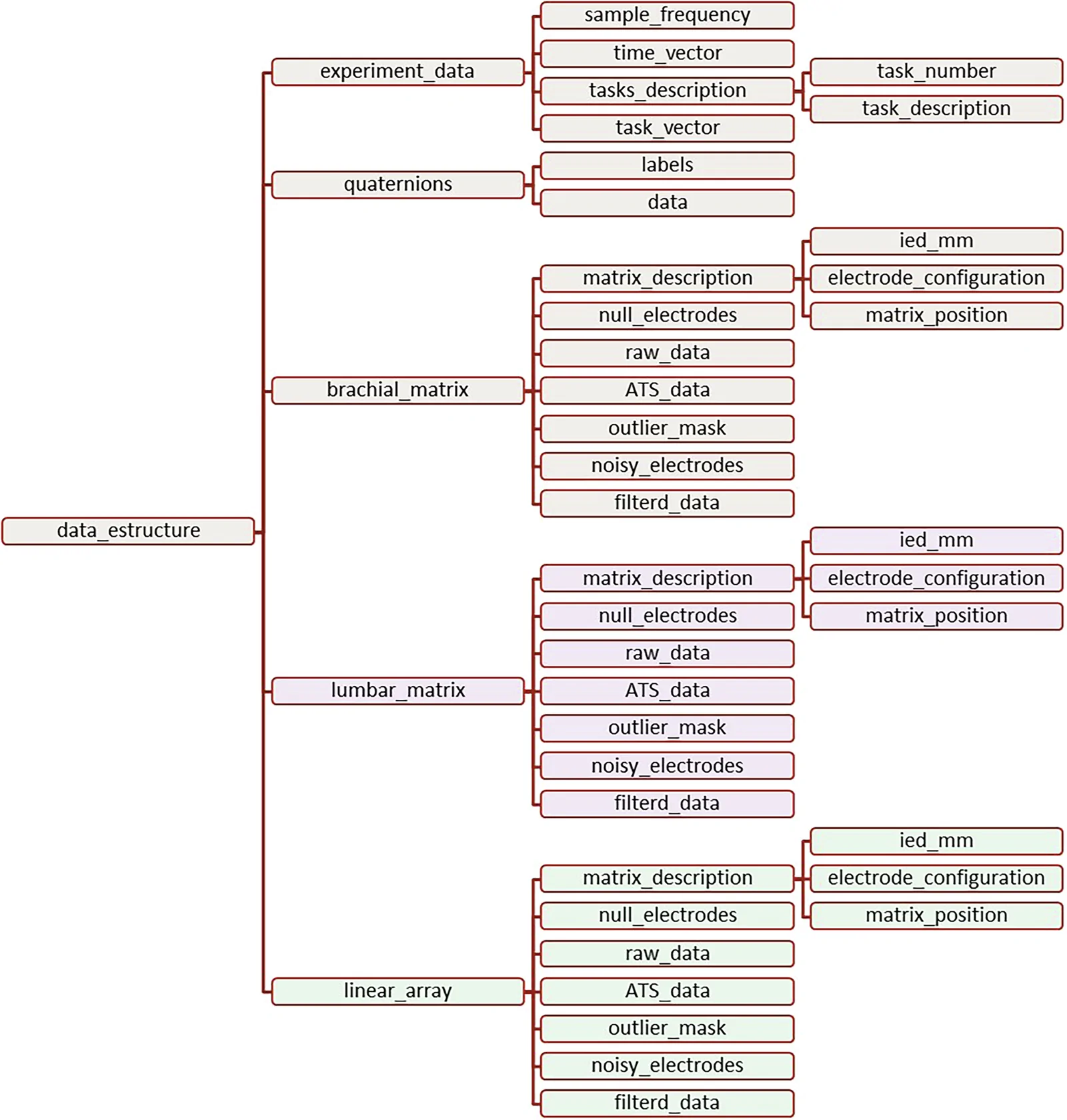
Fields of the MATLAB structure storing data for each trial. Legend: Fields highlighted in brown are present in all experiments, those in pink are specific to the second experiment and those in green are exclusive to the third experiment.

Each MATLAB file contains a structure array, named *data_structure*, with all relevant information for each trial. The fields of this structure are summarized in Fig. 3 and detailed below:

- *experiment_data*: Metadata about the experiment, including:

–*sample_frequency*: Sampling frequency of the recordings.

– *time_vector*: Time vector (seconds).

–*tasks_description*: Structure describing task numbers and their descriptions.

– *task_vector*: Vector indicating the task assigned to each sample.

- *quaternions*: Spatial orientation data, including:

–*labels*: Labels for each recorded quaternion.

–*data*: Matrix containing quaternion data (quaternions  × samples).

- *brachial_matrix* (present in all experiments):
- *matrix_description*: Information about the matrix design, including:

* *ied_mm*: Inter-electrode distance in millimeters.

* *electrode_configuration*: Layout of the electrode matrix as seen on the participant’s back.

* *matrix_position*: Reference point used to position the matrix.

– *null_electrodes*: Binary vector indicating null electrodes (1 for null). Some electrodes experienced connection issues and were registered with a value of 0 throughout the entire recording, these electrodes were considered null. During preprocessing, these electrodes were marked as NaN, as they were not analyzable.

– *raw_data*: Raw data matrix (electrodes  × samples) in microvolts (*μV*).

– *ATS_data*: Preprocessed data matrix (electrodes  × samples) in *μV*.

– *outlier_mask*: Binary matrix (electrodes  × samples) indicating outlier samples (1 for outlier), the process employed to classify the outliers is explained in Section Technical Validation.

– *noisy_electrodes*: Binary vector indicating noisy electrodes (1 for noisy), the process employed to classify the noisy electrodes is explained in Section Technical Validation.

– *filtered_data*: ATS filtered data matrix (electrodes  × samples) after substituting the outlier samples and noisy electrodes samples with NaN values, in *μV*. This variable has not been included in the repository due to storage limitations. However, it can be generated by setting *flag_filtered_data* to TRUE and disabling other unnecessary flags in the provided code^18^.

- *lumbar_matrix* (present only in the second experiment):

– Contains the same fields as the *brachial_matrix*: *matrix_description* (*ied_mm*, *electrode_configuration* and *matrix_position*), *null_electrodes*, *raw_data*, *ATS_data*, *outlier_mask*, *noisy_electrodes* and *filtered_data*.

- *linear_array* (present only in the third experiment):

– Contains the same fields as the *brachial_matrix*: *matrix_description* (*ied_mm*, *electrode_configuration* and *matrix_position*), *null_electrodes*, *raw_data*, *ATS_data*, *outlier_mask*, *noisy_electrodes* and *filtered_data*.

## Technical Validation

The recorded signals were acquired from surface electrode matrices placed on participants’ trunks, which inherently led to the presence of cardiac artifacts. The morphology and characteristics of the QRS complex within these artifacts are influenced not only by the positioning of the electrode matrix–primarily its distance from the heart and the reference electrode–but also by individual subject variability. However, this variability is accounted for through the application of the ATS algorithm, which iteratively adapts to each heartbeat to ensure effective denoising.

**Fig. 4:**
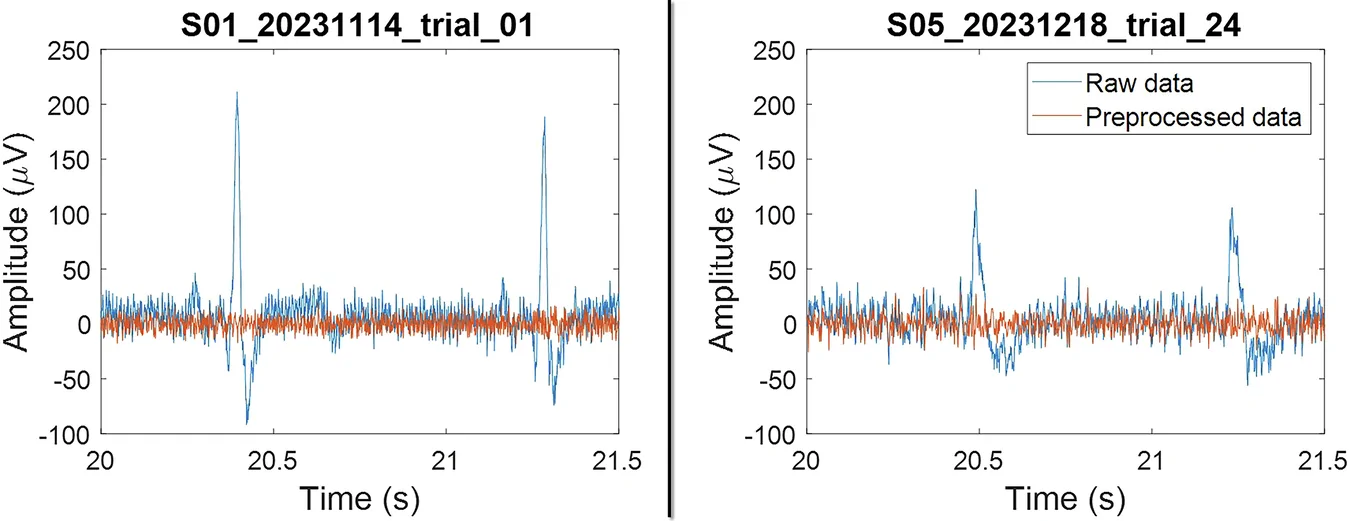
Comparison of the ESG signal after filtering with the ATS algorithm, illustrating the first electrode of the brachial matrix during a trial of stage 1 for subject S01 on the first experiment and subject S05 on the second experiment. The reductions in Kurtosis Ratio (KR2) values between the raw and ATS-filtered signals were 98% and 94%, respectively.

Figure 4 presents two examples of signals recorded from the brachial matrix, each exhibiting distinct QRS complex morphologies but, in both cases, the ATS-filtered signal demonstrates effective artifact removal. To quantify this effect it was employed the Kurtosis Ratio (KR2), a robust kurtosis-based metric proposed as an indicator of ECG contamination in EMG recordings^19^. This metric evaluates the distribution of the estimated signal relative to a standard Gaussian distribution, with higher absolute KR2 values reflecting a greater degree of ECG interference^19^.

Validation of the ATS algorithm for the removal of cardiac artifacts in ESG signals showed a mean reduction exceeding 90% in KR2 when comparing raw and filtered signals^15^. In the examples shown in Fig. 4, the KR2 values of the raw signals were 2.65 and 1.91, respectively. After applying the ATS algorithm, these values were reduced to 0.04 and 0.11, corresponding to reductions of 98% and 94%, respectively. These results confirm the algorithm’s strong capability to mitigate cardiac interference across varying signal morphologies.

**Fig. 5:**
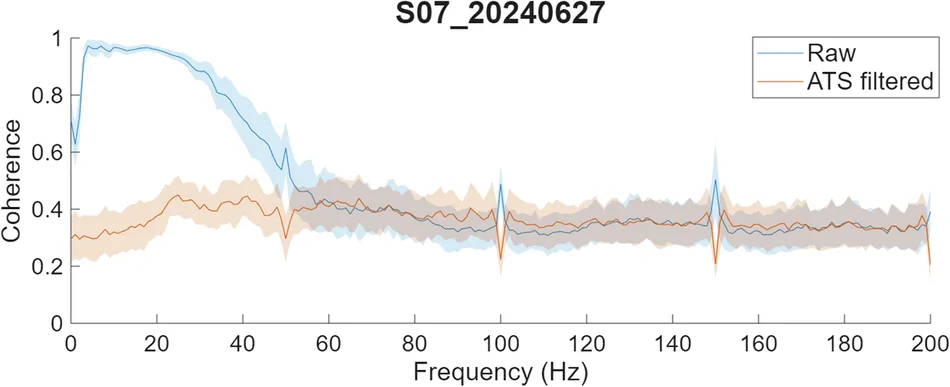
Mean coherence values and their corresponding standard deviations across frequency bands between signals recorded from the brachial and lumbar plexus under two conditions: raw signals and ATS-filtered signals. The results correspond to the trials recorded for the second experiment of subject S07.

Coherence analysis across different frequency bands was also performed to assess the relationship between signals recorded from the brachial and lumbar plexus matrices before and after ATS filtering. Because both locations capture spinal cord activity at different rostrocaudal levels, some degree of correlation is expected, though not necessarily of high magnitude. As shown in Fig. 5, coherence values prior to denoising were close to 1 at frequencies dominated by cardiac artifacts. After ATS filtering, coherence became more uniform across the spectrum, demonstrating that the denoising procedure effectively removed shared cardiac components and revealed underlying neural activity with more consistent inter-matrix relationships.

**Fig. 6:**
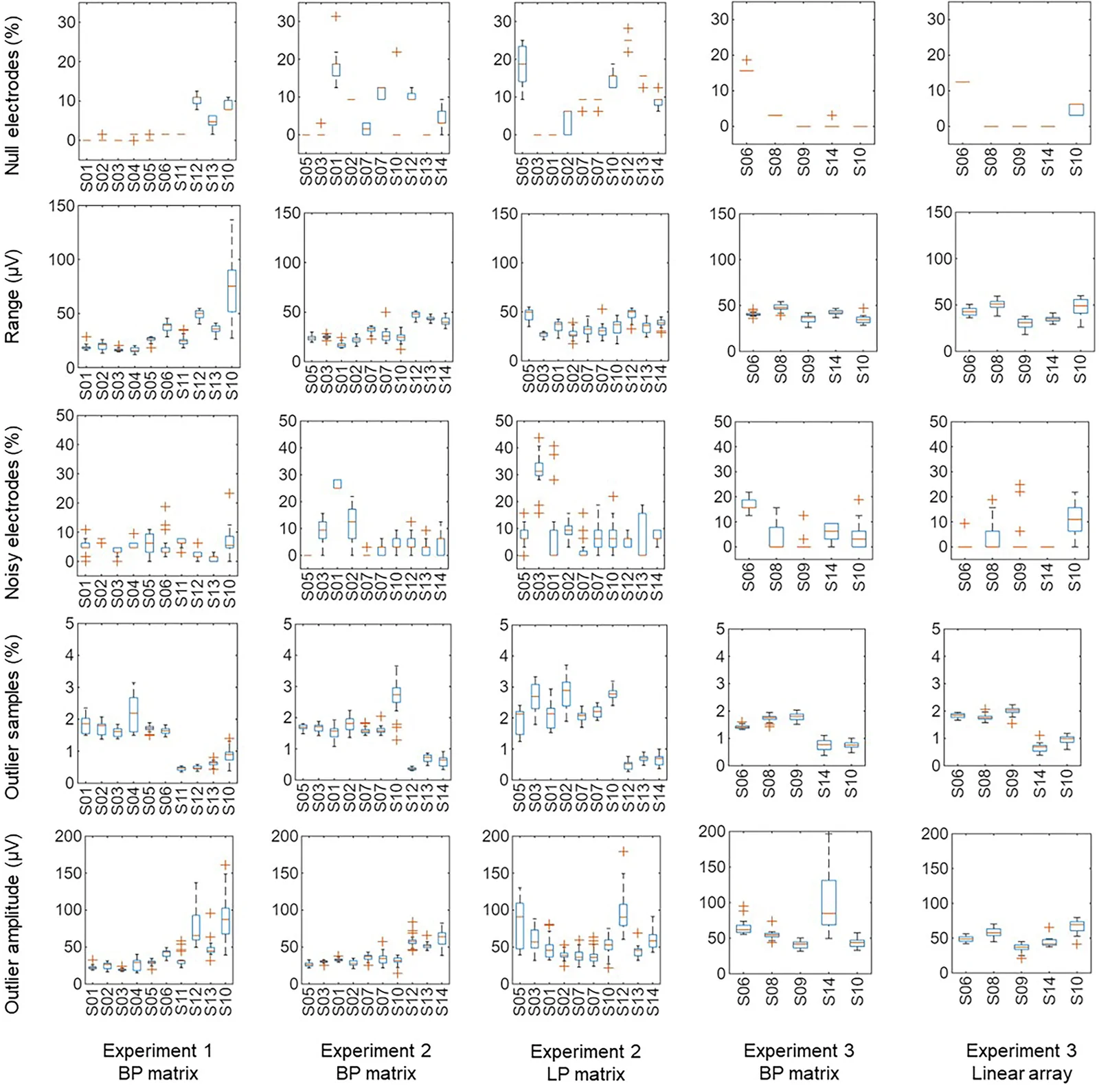
The metrics presented in Table 2, together with their corresponding confidence intervals, are illustrated using boxplots for each metric and for each group of subjects who performed the experiments, with results stratified according to the matrix configuration employed. The metrics considered include the ratio of null and noisy electrodes, the proportion of outlier samples, the mean range and the outlier amplitude. All metrics were computed at the level of individual electrodes and trials and averaged values are reported for each matrix configuration.

Although the primary components of cardiac artifacts were effectively filtered, residual cardiac and non-cardiac artifacts may persist. To characterize the resulting signal quality after all preprocessing steps, a set of quantitative metrics was computed across subjects, trials and electrode matrices. These metrics include mean signal amplitude, the percentage of electrodes classified as null or noisy and the proportion of samples identified as outliers along their mean amplitude. Table 2 summarizes these aggregated values, providing a concise overview of signal quality across experiments. Complementing these statistics, Fig. 6 presents boxplot distributions for each metric, enabling visual assessment of inter-subject variability and highlighting distributional properties not captured by summary statistics alone.

**Table 2:** Metrics, presented as mean  ± standard deviation, including the ratio of null and noisy electrodes, the proportion of outlier samples, mean range and outlier amplitude, obtained for each subject and experiment.

Experiment 1 - Brachial Matrix					
**Subject**	**Null electrodes (%)**	**Range (*μV*)**	**Noisy electrodes (%)**	**Outlier samples (%)**	**Outlier amplitude (*μV*)**
S01_20231114	0.00 ± 0.00	18.78 ± 2.46	4.76 ± 2.56	1.85 ± 0.28	22.35 ± 3.03
S02_20231115	0.07 ± 0.32	20.05 ± 3.51	7.75 ± 0.32	1.73 ± 0.20	24.80 ± 4.42
S03_20231204	0.00 ± 0.00	16.33 ± 1.41	3.32 ± 1.55	1.61 ± 0.14	19.49 ± 1.62
S04_20231205	1.30 ± 0.59	16.21 ± 2.27	5.47 ± 1.13	2.20 ± 0.52	27.00 ± 7.59
S05_20231218	0.07 ± 0.33	26.12 ± 2.40	5.91 ± 3.52	1.72 ± 0.10	29.63 ± 3.39
S06_20231219	1.56 ± 0.00	37.73 ± 4.67	4.95 ± 4.34	1.63 ± 0.10	41.30 ± 5.04
S11_20250220	1.56 ± 0.00	24.63 ± 4.23	6.32 ± 1.26	0.45 ± 0.06	32.71 ± 9.07
S12_20260305	10.48 ± 1.34	49.23 ± 4.17	2.15 ± 1.29	0.45 ± 0.06	75.69 ± 24.19
S13_20260305	4.69 ± 1.60	35.34 ± 3.95	1.24 ± 1.13	0.62 ± 0.09	48.05 ± 11.90
S10_20260312	8.92 ± 1.34	75.15 ± 27.15	6.97 ± 4.85	0.87 ± 0.23	89.34 ± 31.03
**Mean**	2.86 ± 0.55	31.96 ± 5.62	4.88 ± 2.19	1.32 ± 0.18	41.04 ± 10.13
**Experiment 2 - Brachial Matrix**					
**Subject**	**Null electrodes (%)**	**Range (*μV*)**	**Noisy electrodes (%)**	**Outlier samples (%)**	**Outlier amplitude (*μV*)**
S05_20240507	0.00 ± 0.00	23.77 ± 2.62	0.00 ± 0.00	1.69 ± 0.08	26.38 ± 2.99
S03_20240507	0.13 ± 0.64	24.93 ± 1.43	9.11 ± 4.96	1.67 ± 0.13	30.27 ± 1.76
S01_20240523	17.71 ± 3.76	16.77 ± 2.72	26.30 ± 1.57	1.54 ± 0.20	32.91 ± 2.07
S02_20240611	9.38 ± 0.00	21.79 ± 3.48	12.11 ± 6.92	1.80 ± 0.25	28.11 ± 4.27
S07_20240627	1.56 ± 1.60	32.45 ± 3.25	0.26 ± 0.88	1.58 ± 0.10	36.35 ± 3.98
S07_20241015	11.01 ± 1.60	26.29 ± 6.81	2.04 ± 1.79	1.61 ± 0.13	34.12 ± 8.26
S10_20250211	0.91 ± 4.47	24.16 ± 4.77	3.91 ± 2.80	2.68 ± 0.54	31.17 ± 5.37
S12_20260309	10.16 ± 1.38	47.19 ± 3.42	4.69 ± 3.19	0.36 ± 0.04	57.90 ± 8.39
S13_20260309	0.00 ± 0.00	43.37 ± 2.38	2.86 ± 2.90	0.70 ± 0.13	51.61 ± 4.29
S14_20260312	4.17 ± 3.01	40.39 ± 4.14	4.43 ± 3.68	0.61 ± 0.16	62.42 ± 12.36
**Mean**	5.50 ± 1.64	30.11 ± 3.50	6.57 ± 2.87	1.42 ± 0.18	39.12 ± 3.57
**Experiment 2 - Lumbar Matrix**					
**Subject**	**Null electrodes (%)**	**Range (*μV*)**	**Noisy electrodes (%)**	**Outlier samples (%)**	**Outlier amplitude (*μV*)**
S05_20240507	18.49 ± 5.82	47.32 ± 6.36	7.55 ± 4.11	1.93 ± 0.41	84.73 ± 30.86
S03_20240507	0.00 ± 0.00	26.51 ± 2.79	32.42 ± 6.50	2.65 ± 0.46	59.15 ± 15.07
S01_20240523	0.00 ± 0.00	35.43 ± 5.62	8.72 ± 14.86	2.12 ± 0.41	48.87 ± 13.95
S02_20240611	3.91 ± 3.09	27.52 ± 4.92	9.64 ± 3.18	2.12 ± 0.41	39.70 ± 6.87
S07_20240627	8.85 ± 1.19	31.30 ± 6.58	2.08 ± 4.19	2.06 ± 0.18	37.42 ± 8.78
S07_20241015	8.70 ± 1.32	30.82 ± 6.77	6.93 ± 5.07	2.20 ± 0.19	38.53 ± 10.38
S10_20250211	14.71 ± 1.72	34.60 ± 7.72	6.90 ± 4.87	2.78 ± 0.18	51.76 ± 14.26
S12_20260309	24.87 ± 1.45	47.76 ± 5.90	5.47 ± 2.30	0.49 ± 0.12	96.52 ± 26.05
S13_20260309	15.49 ± 0.64	34.46 ± 5.98	4.95 ± 7.42	0.69 ± 0.10	43.92 ± 8.07
S14_20260312	9.24 ± 2.16	38.57 ± 3.98	7.16 ± 2.35	0.64 ± 0.18	59.67 ± 12.67
**Mean**	10.43 ± 1.74	35.43 ± 5.66	9.18 ± 5.49	1.84 ± 0.27	56.03 ± 14.07
**Experiment 3 - Brachial Matrix**					
**Subject**	**Null electrodes (%)**	**Range (*μV*)**	**Noisy electrodes (%)**	**Outlier samples (%)**	**Outlier amplitude (*μV*)**
S06_20241004	15.76 ± 0.64	40.52 ± 2.06	16.54 ± 2.35	1.42 ± 0.07	65.44 ± 10.74
S08_20250210	3.12 ± 0.00	47.85 ± 3.86	3.91 ± 5.31	1.73 ± 0.12	54.61 ± 5.78
S09_20250210	0.00 ± 0.00	35.40 ± 4.01	0.65 ± 2.60	1.78 ± 0.14	40.82 ± 5.22
S14_20260312	0.26 ± 0.88	42.75 ± 2.46	5.86 ± 3.61	0.76 ± 0.20	102.02 ± 44.39
S10_20260317	0.00 ± 0.00	34.51 ± 4.3	3.39 ± 4.60	0.74 ± 0.15	43.20 ± 6.14
**Mean**	3.83 ± 0.30	40.20 ± 3.34	6.07 ± 3.69	1.29 ± 0.14	61.22 ± 14.46
**Experiment 3 - Linear Matrix**					
**Subject**	**Null electrodes (%)**	**Range (*μV*)**	**Noisy electrodes (%)**	**Outlier samples (%)**	**Outlier amplitude (*μV*)**
S06_20241004	12.50 ± 0.00	43.04 ± 4.37	0.39 ± 1.91	1.83 ± 0.09	48.92 ± 4.50
S08_20250210	0.00 ± 0.00	50.63 ± 5.59	3.65 ± 6.43	1.77 ± 0.11	58.60 ± 6.83
S09_20250210	0.00 ± 0.00	30.21 ± 5.59	4.04 ± 8.62	2.00 ± 0.15	36.11 ± 5.88
S14_20260312	0.00 ± 0.00	34.92 ± 3.05	0.00 ± 0.00	0.66 ± 0.17	43.70 ± 5.94
S10_20260317	5.34 ± 1.45	47.53 ± 8.73	10.55 ± 6.70	0.94 ± 0.18	66.87 ± 9.23
**Mean**	3.57 ± 0.29	41.26 ± 5.46	3.72 ± 4.73	1.44 ± 0.14	50.84 ± 6.48

These metrics were computed for each electrode and trial, with averages provided for each matrix configuration.

**Fig. 7:**
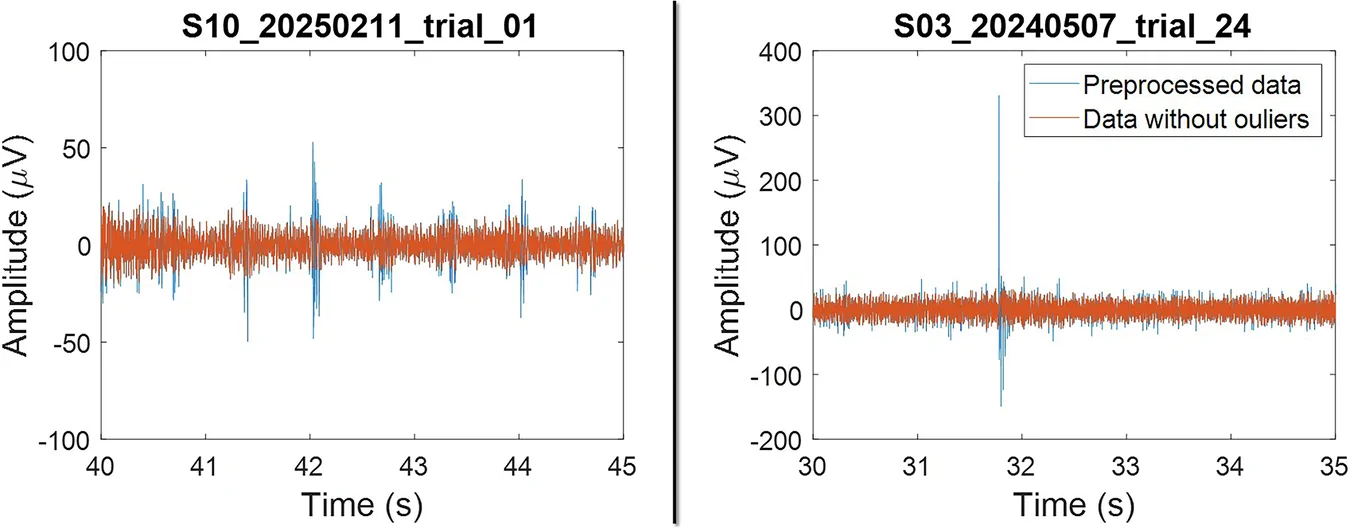
Comparison of the ESG signal after outlier elimination, representing the first electrode of the lumbar matrix during a trial of stage 1 for subject S03 and S10 on the second experiment. The reductions in Kurtosis Ratio (KR2) values between the ATS-filtered signals and the outlier-eliminated signals were 71% and 83%, respectively. Note: the axis limits are adjusted to better represent the residual ECG complex in the first case and the artifact presence in the second one.

Figure 7 provides an example of both artifact types along with the outcomes of an outlier elimination procedure implemented to address these issues. This procedure involved segmenting the data into half-second intervals and calculating a modified z-score for each segment, with each electrode analyzed independently. The modified z-score was computed using the median absolute deviation (MAD), a more robust statistical approach. Outliers exceeding a threshold of 3 were identified and replaced with NaN values to maintain data integrity for subsequent analyses.

The presence of NaN values may limit certain temporal analyses, particularly those requiring continuous signals. This limitation can be mitigated by applying window-based processing and averaging results across windows that do not contain NaN samples. When such approaches are not feasible, the variable *ATS_data* may be used instead, as it contains no NaN values; however, it may still include residual artifacts that could influence specific analyses.

In the examples shown in Fig. 7, the KR2 values of the raw signals were 11.51 and 0.49, respectively. After applying the ATS algorithm, these values were reduced to 0.69 and 0.16. Although this represents a substantial reduction, the resulting KR2 values remain higher than those obtained in other examples. Following the outlier-elimination procedure, the KR2 values were further reduced to 0.20 and −0.03, corresponding to reductions of 71% and 83%, respectively, relative to the ATS-filtered signals.

The mean signal amplitude of the resulting signals was approximately 30 *μ*V across all matrices, see Table 2 for individual values. These values align with the findings of Steele *et al*.^12^, who recorded ESG signals using surface electrodes. The amplitude of ESG signals varies significantly based on the recording technique: more invasive methods, such as epidural or intradural recordings^9,10^, generally exhibit higher signal amplitudes, while surface-level recordings, as employed in this study, produce lower amplitudes^20^. Direct comparisons with existing literature remain challenging due to methodological differences in signal processing, as much of the available research focuses on evoked potentials^9–11^.

Following outlier elimination, noisy electrodes were identified and excluded from further analysis. This process involved calculating the mean range of each segment, averaging across all segments within a trial, and computing a modified z-score for each electrode. Electrodes with z-scores exceeding 3 were classified as noisy and replaced with NaN values. The thresholds were selected based on an exploratory parameter-scanning procedure. A threshold of 3.5 was found to be inadequate, as it failed to capture infrequent but meaningful outliers, mainly cardiac artifacts remains, and therefore did not provide sufficient denoising. Conversely, a threshold of 2.5 behaved more like a hard-thresholding operation, disproportionately suppressing temporally localized regions that exhibited consistently higher-amplitude yet physiologically valid signals. In contrast, a threshold of 3 offered the most appropriate balance, effectively distinguishing true outliers from legitimately high-percentile signal amplitudes. Across all experiments, an average of 6.38% of electrodes were identified as noisy, see Table 2 for all individual values.

**Fig. 8:**
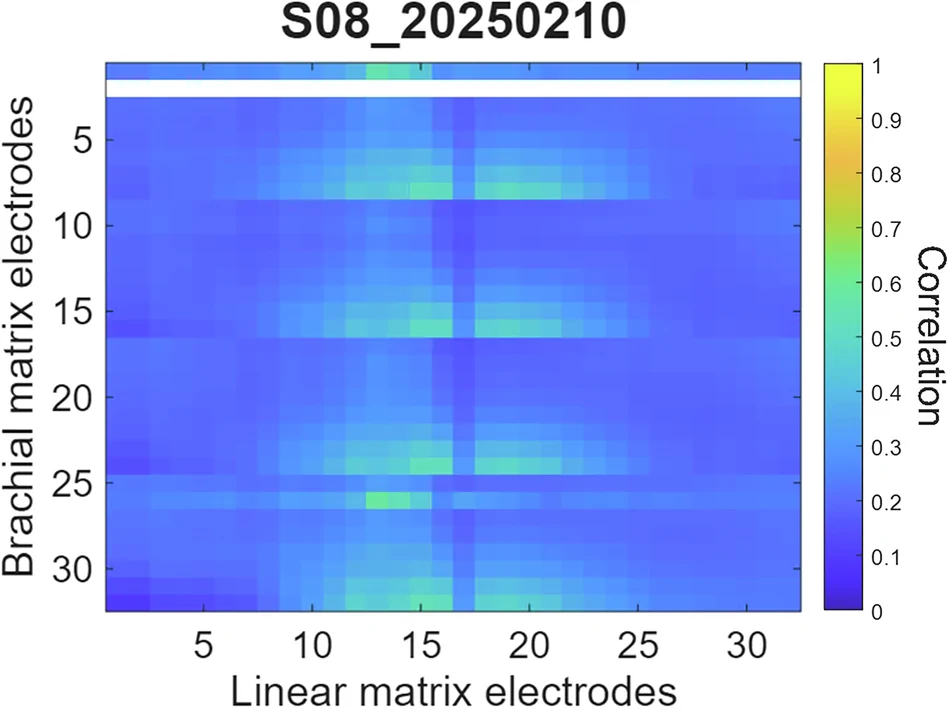
Average correlation, expressed as a proportion, between the brachial matrix and the linear array among the trials for the third experiment of subject S08. Electrodes classified as null or noisy are shown in white.

To verify the independence of signals recorded from surface electrode matrices and to ensure no unintended connections existed between electrodes, intra-matrix correlations were analyzed. Correlations were computed over one-second windows for each subject and trial, then averaged. The mean correlation was 69.95%, with values ranging from −37.57% to 99.41%. Variability in correlation was influenced by the inter-electrode distance, with higher correlations observed between electrodes in close proximity. These spatial trends can be observed in Fig. 8. Notably, some negative correlations were identified between electrodes located at opposing corners of the linear array. Overall, these findings confirm the expected spatial structure within a densely spaced electrode matrix: although the underlying signal source is deep and its activity propagates across all surface electrodes, measurable differences persist and diminish with decreasing inter-electrode distance. These observations also have implications for spatial filtering techniques such as Laplacian filters, which may enhance electrode independence, but could also suppress common components of the signal, including deep spinal activity.

To further investigate potential interference in the recorded signals, the third experiment was conducted using a linear electrode array spanning 15.5 cm, positioned just below the brachial matrix. This array aimed to capture signals originating from both the spinal cord and surrounding musculature. The hypothesis was that muscles at different depths, particularly those involved in maintaining an upright posture, are activated during gait and their activation could be detected by the recording device. Correlation analysis between the signals recorded by this array and the registered quaternions yielded values in the range of [−0.0096, 0.0087], indicating minimal linear association. Notably, this array underwent the same preprocessing steps as the brachial matrix, including high-pass filtering at 20 Hz, which substantially attenuates low-frequency components such as cardiac and slow movement-related activity. Because quaternions primarily encode orientation changes occurring below approximately 1 Hz, most motion-related interference present in the raw signals would be strongly reduced by this filtering step. Nonetheless, while these results suggest limited coupling between motion and the preprocessed signals, they do not fully exclude the possibility of residual motion artifacts.

**Fig. 9:**
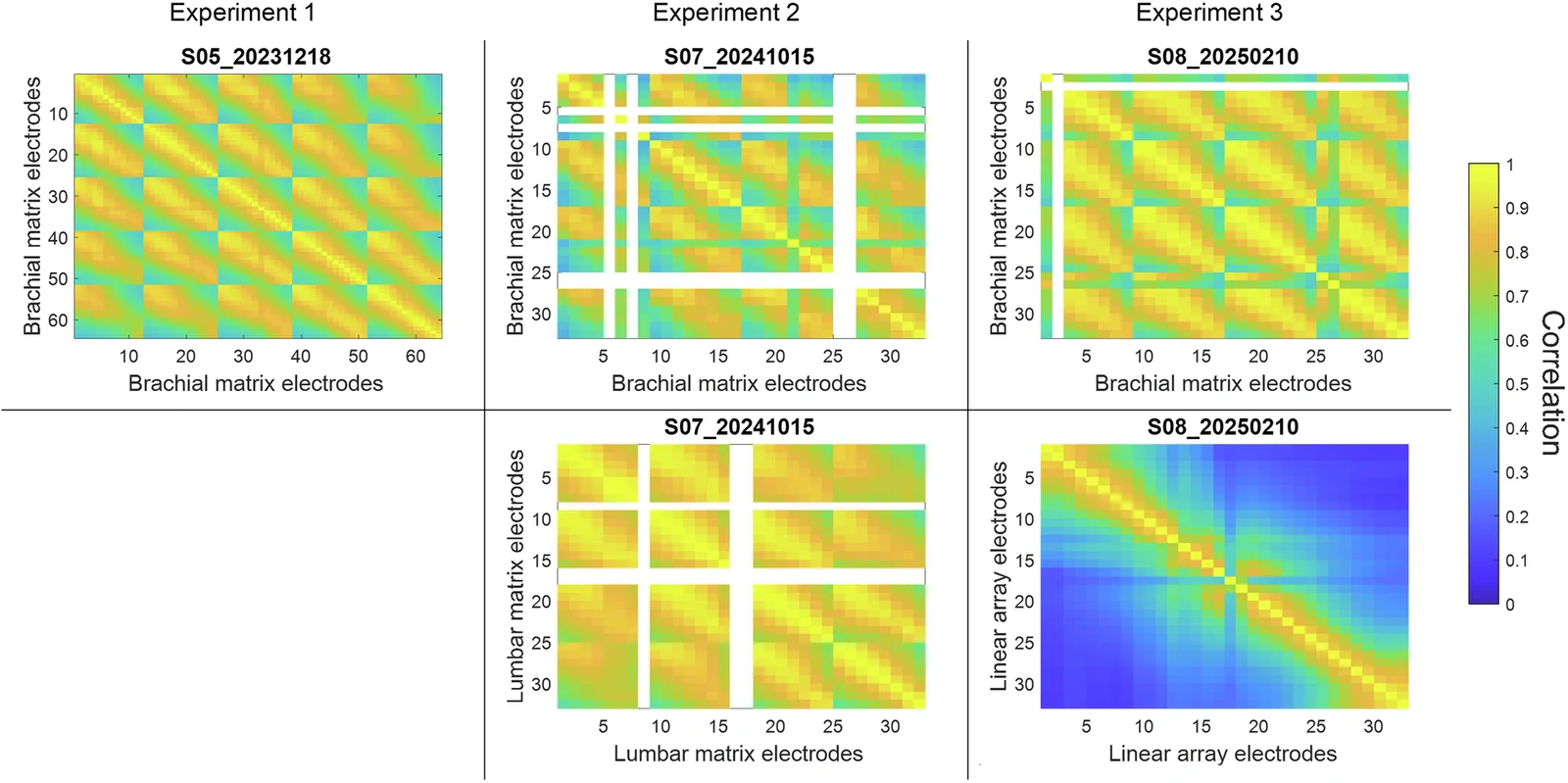
Average correlation, expressed as a proportion, between different matrix configuration, exemplifying a subject for each experiment. Electrodes classified as null or noisy are shown in white.

The correlation between the brachial matrix and the linear array was also examined. Higher correlations were observed in central electrodes positioned directly above the spine, with amplitudes decreasing as the electrode distance increased, as seen in Fig. 9. Electrodes located farther from the spinal column exhibited lower correlations, with a few cases of negative correlation of the electrodes position on the left side of the back.

To evaluate the correlation between the brachial matrix and the linear array across the static and movement conditions of the stage 3 trials, a statistical analysis was conducted. The dataset included recordings from 5 subjects, each performing five trials under static and movement conditions. The correlation values between electrode matrices were computed for each trial, resulting in a dataset of correlation coefficients.

To assess the normality assumption of each sample, the Shapiro-Wilk and Shapiro-Francia tests were employed based on the kurtosis range of each sample. It was determined that the samples did not exhibit a normal distribution (p > 0.01). As a result, non-parametric statistical methods were selected for further analysis.

A Kruskal-Wallis test was performed to examine potential subject dependency and the results indicated no statistically significant differences among the subjects (p > 0.05), confirming that the data could be pooled for further comparison between conditions.

To investigate differences between static and movement conditions, a Kruskal-Wallis test was performed on the pooled dataset. The results indicated no statistically significant difference between the two conditions (p > 0.01). This finding suggests that the correlation patterns between the the brachial matrix and the underlying linear array are not influenced by the nature of the activity, whether static or movement. It is important to reiterate that the linear array also captured activity from the musculature surrounding the spinal column. If increased EMG activity had been recorded during the dynamic condition, and if such activity had produced measurable interference in the brachial matrix, the resulting correlation values would likely have differed significantly from those observed in the static condition. Instead, the absence of significant differences suggests that the musculature recorded was only minimally activated during walking and that any EMG interference affecting the brachial matrix was negligible.

In summary, the technical validation procedures confirmed the reliability and quality of the recorded ESG signals. Despite inherent variability and the presence of both cardiac and non-cardiac artifacts, the application of ATS filtering and robust outlier detection enabled effective signal denoising. Correlation analyses revealed expected spatial trends, supporting electrode independence and accurate matrix placement. Furthermore, statistical comparisons between static and dynamic conditions revealed no significant differences in the correlation values between the brachial matrix and the underlying linear array, reinforcing the conclusion that direct motion-related EMG interference was absent or minimal. Collectively, these findings support the robustness and reproducibility of the data acquisition and preprocessing pipeline.

## Usage Notes

The MATLAB script available on GitHub^18^ is designed to analyze all database files related to preprocessing and technical validation processes.

The core function, *process_signal*, consists of two specialized subfunctions, each dedicated to a specific aspect of signal preprocessing:

- *ATS_filter_signals*: Performs ECG denoising and stores the processed data in the *ATS_data* (see Section Data Format). Proper operation of this function requires the inclusion of Petersen’s cardiac artifact removal toolbox^14^.
- *compute_noisy_outliers*: Identifies outlier samples and detects noisy electrodes using the variables detailed in Section Data Format.

The outputs generated by these functions have been precomputed and are included in the provided database.

The technical validation process is performed through three dedicated functions:

- *compute_metric_table*: Generates a table containing key quality metrics, as described in Section Technical Validation. These metrics include the ratio of null and noisy electrodes, the proportion of outlier samples, mean range and outlier amplitude. The results are stored in a matrix structured as (metrics, subject, experiment, trial), where different matrix configurations are distinguished and values are averaged across electrodes.
- *compute_correlation_time*: Calculates a correlation matrix representing the correlation coefficients among electrodes from different configurations depending on the experiment and the quaternions. The results are stored in a matrix formatted as (electrodes, electrodes, subject, experiment, trial), with values averaged across window segments. The electrode configurations for each experimental setup are as follows:

– Experiment 1: 64 electrode of the brachial matrix and 4 quaternions.

– Experiment 2: 32 electrode of the brachial matrix, 32 electrodes of the lumbar matrix and 4 quaternions.

– Experiment 3: 32 electrode of the brachial matrix, 32 electrodes of the matrix, linear array and 4 quaternions.

- *compute_coherence*: Calculates a coherence matrix representing the coherence value across the frequency bins before and after the filtering performed by the ATS algorithm. The results are stored in a matrix with dimensions (frequencies, condition, subject, test, trial). This function is applied exclusively to the data from the second experiment, computing the coherence between signals recorded from the brachial and lumbar plexuses.

Additionally, the *comb_filter* function has been implemented to perform bandpass filtering, along with multiple notch filters, using a state-variable filter design. This function is supported by auxiliary functions (*read_func*, *state_filter*, *state_filter_sample* and *filter_setup*) which modularize the filtering process and enhance code organization.

To allow flexibility in processing, a set of flags has been incorporated, enabling selective execution of specific steps:

- *flag_ATS*: Executes preprocessing steps and applies ATS denoising.
- *flag_outlier*: Identifies outlier samples and noisy electrodes.
- *flag_filtered_data*: Updates the data matrix by replacing values of outlier samples and noisy electrodes with NaNs.
- *flag_metrics*: Computes the quality metrics.
- *flag_correlation*: Computes the correlation between quaternion and electrode data.
- *flag_coherence*: Computes the coherence between two matrix of electrode data.
- *flag_save*: Saves the updated *data_structure* variable.

The files have been tested on MATLAB R2023a for the uploaded software version.

## Data Availability

The datasets are publicly available on Zenodo^17^.

## Code availability

The MATLAB script is available on GitHub^18^.
